# Sick Headache

**Published:** 1872-10

**Authors:** 


### SICK HEADACHE

Almost always depends upon an ill condition of the stomach from over-eating, and may be promptly relieved by drinking a tea-cup full of water, a little warm, every five minutes, until the stomach is so full of water that a feather or finger in the throat will give an effectual discharge of all the stomach contains.

If it is simply a nervous affection, sit in hot water for fifteen minutes; the good effects are sometimes increased if some carbonate of soda is put in the water, one ounce to two gallons. The philosophy of the action of the water is that it draws the excess of blood, which oppresses the interior organs, to the surface.

